# MRgFUS of osteoid osteoma started at the Rizzoli

**DOI:** 10.1186/2050-5736-2-S1-A14

**Published:** 2014-12-10

**Authors:** Alberto Bazzocchi, Alessandro Napoli, Paolo Spinnato, Danila Diano, Giancarlo Facchini, Maurizio Busacca, Carlo Catalano, Ugo Albisinni

**Affiliations:** 1 Diagnostic and Interventional Radiology, The “Rizzoli” Orthopaedic Institute, Bologna, 40136, Italy; 2 Department of Specialized, Diagnostic, and Experimental Medicine, University of Bologna, Bologna, 40138, Italy; 3 Department of Radiological, Oncological and Anatomo-pathological Sciences, “Sapienza” University of Rome, Rome, 00161, Italy

## Background

Osteoid osteoma is a bone-producing lesion frequently localized in long bones. The most common presenting symptom is pain with nocturnal exacerbation, responsive to nonsteroidal anti-inflammatory drugs [1]. Minimally invasive treatment options have become the standard of care for osteoid osteoma and at present radiofrequency ablation is the treatment of choice [2]. Only one preliminary study has been performed using magnetic resonance guided focused ultrasound (MRgFUS) to treat osteoid osteoma showing MRgFUS to be effective for pain relief through thermal cell death and periosteal denervation induced by cortical heating relative to acoustic energy absorption [3]. The purpose of this research was to evaluate the efficacy of MRgFUS in treating osteoid osteoma and to highlight potential benefits and limitations provided by this technology.

## Materials and methods

Seven patients affected by osteoid osteoma were consecutively recruited for MRgFUS (ExAblate 2100, InSightec, Israel on 1.5 T Signa Twin Speed MR system, GE, USA). The diagnosis of osteoid osteoma was based on typical clinical and imaging findings (CT and MRI) . Pain and function were scored before and after MRgFUS (VAS – 0-10 scale), and any intra- and post-procedure complication or adverse effect was recorded. Initial follow-up included clinical assessment at discharge, and every month.

## Results

In one patient the lesion could not be approached due to the high body mass index (33 Kg/m^2^). This condition did not allow a correct positioning of the patient to find the optimal ultrasound beam window. Six males, ageing 18-46 years old (mean 26 year-old; BMI 26.2±4.9, range 19.3-34.5 Kg/m^2^) and presenting with pain scoring 5 to 10 (7.1±2.1) underwent MRgFUS (Figure 2). All osteoid osteomas were located at femur (head/neck in 4, diaphysis in 2), and in one case the treatment was proposed for recurrence after being treated by radiofrequency. All treatments were performed under spinal anesthesia. The day after the procedure VAS score was 2.3±1.6 (0-5) (p<0.001), and dropped to 0 in all patients from the 1-month check onward. Table 1 reports the detailed data for each patient.

**Table 1:** Functional impairment substantially improved in all patients during the first month, and patients’ status turned to complete restoration of normal function and daily activities. The follow-up is still limited at 3 months.

	n°sonication	mean energy	VASpre	VASpost*	VASpost**	VASpost***
**Patient 1**	5	1855 J	5	0	0	0

**Patient 2**	6	1110 J	8	0	0	0

**Patient 3**	6	1890 J	8	0	0	0

**Patient 4**	6	1900 J	9	0	0	0

**Patient 5**	5	535 J	5	0	0	0

**Patient 6**	8	1650 J	10	5	0	0

* 1 day after the treatment

** 1-month after the treatment

*** 3-month after the treatment

## Conclusion

MRgFUS of osteoid osteoma is an extremely promising treatment. Our first experience confirms its safety and efficacy. Additional imaging evaluation and an extended follow-up will be able to support preliminary evidence and to examine the biological evolution of the bone lesion.
